# Alveolar macrophage-epithelial cell interaction following exposure to atmospheric particles induces the release of mediators involved in monocyte mobilization and recruitment

**DOI:** 10.1186/1465-9921-6-87

**Published:** 2005-08-01

**Authors:** Hiroshi Ishii, Shizu Hayashi, James C Hogg, Takeshi Fujii, Yukinobu Goto, Noriho Sakamoto, Hiroshi Mukae, Renaud Vincent, Stephan F van Eeden

**Affiliations:** 1 James Hogg *i*CAPTURE Centre for Cardiovascular and Pulmonary Research, St. Paul's Hospital, University of British Columbia, 1081 Burrard Street, Vancouver, BC, V6Z 1Y6, Canada; 2 Second Department of Internal medicine, Nagasaki University School of Medicine, Nagasaki, Japan; 3 Environmental Health Directorate, Health Canada, Ottawa, Ontario, Canada

## Abstract

**Background:**

Studies from our laboratory have shown that human alveolar macrophages (AM) and bronchial epithelial cells (HBEC) exposed to ambient particles (PM_10_) *in vitro *increase their production of inflammatory mediators and that supernatants from PM_10_-exposed cells shorten the transit time of monocytes through the bone marrow and promote their release into the circulation.

**Methods:**

The present study concerns co-culture of AM and HBEC exposed to PM_10 _(EHC-93) and the production of mediators involved in monocyte kinetics measured at both the mRNA and protein levels. The experiments were also designed to determine the role of the adhesive interaction between these cells via the intercellular adhesion molecule (ICAM)-1 in the production of these mediators.

**Results:**

AM/HBEC co-cultures exposed to 100 μg/ml of PM_10 _for 2 or 24 h increased their levels of granulocyte-macrophage colony-stimulating factor (GM-CSF), M-CSF, macrophage inflammatory protein (MIP)-1β, monocyte chemotactic protein (MCP)-1, interleukin (IL)-6 and ICAM-1 mRNA, compared to exposed AM or HBEC mono-cultures, or control non-exposed co-cultures. The levels of GM-CSF, M-CSF, MIP-1β and IL-6 increased in co-cultured supernatants collected after 24 h exposure compared to control cells (p < 0.05). There was synergy between AM and HBEC in the production of GM-CSF, MIP-1β and IL-6. But neither pretreatment of HBEC with blocking antibodies against ICAM-1 nor cross-linking of ICAM-1 on HBEC blocked the PM_10_-induced increase in co-culture mRNA expression.

**Conclusion:**

We conclude that an ICAM-1 independent interaction between AM and HBEC, lung cells that process inhaled particles, increases the production and release of mediators that enhance bone marrow turnover of monocytes and their recruitment into tissues. We speculate that this interaction amplifies PM_10_-induced lung inflammation and contributes to both the pulmonary and systemic morbidity associated with exposure to air pollution.

## Background

Exposure to ambient particulate matter with a diameter of less than 10 μm (PM_10_) is strongly associated with increased morbidity and mortality, particularly in subjects with pre-existing pulmonary and cardiovascular diseases [1,2]. This increase in mortality induced by PM_10 _exposure was present even when adjusted for the other major risk factors such as cigarette smoking [1]. A recent report [3] has shown that environmentally relevant concentrations of PM_2.5 _induced airway inflammation even in healthy subjects with a selective influx of monocytes.

Although the biological mechanisms are still unclear, PM_10 _are known to stimulate the production of reactive oxygen species and inflammatory mediators by alveolar macrophages (AM) [4-7] and epithelial [7-10] and other lung cells [11]. When AM and airway epithelial cells are directly exposed to inhaled atmospheric particles these small particles are phagocytized by both cells [10,12]. Both cell types can synthesize a variety of pro-inflammatory cytokines that induce airway inflammation and contribute to the airway lesions in asthma and chronic obstructive pulmonary diseases [9]. *In vitro*, AM and lung epithelial cells interact in response to PM_10 _and this interaction has been implicated in amplifying their mediator production [7,13]. Studies from our laboratory have shown that the PM_10_(EHC-93)-induced interaction of human AM and bronchial epithelial cells (HBEC) enhances the synthesis and release of a variety of pro-inflammatory cytokines and that supernatants from these co-cultures instilled into rabbit lungs induces a systemic inflammatory response [13].

We recently showed that deposition of PM_10 _(EHC-93 and inert carbon particles) in the lung shortened the transit time of monocytes through the bone marrow and enhanced their release into the circulation [14,15]. Furthermore, we also showed that monocytes are the predominant inflammatory cells that accumulate in the alveoli following repeated PM_10 _exposure [16]. The present study was designed to determine whether, and if so, which interactions between AM and HBEC (AM/HBEC co-cultures) amplify the response to PM_10 _exposure, especially the synthesis of inflammatory mediators that enhance bone marrow turnover of monocytes and their recruitment into the lung. We used primary cultures of HBEC and human AM freshly isolated from lobectomy or pneumonectomy specimens and measured the expression of inflammatory mediators relevant to monocyte kinetics. We further evaluated the potential role of the intercellular adhesion molecule (ICAM)-1 in the production of mediators by AM/HBEC co-cultures exposed to PM_10_.

## Methods

### Urban air particles (PM_10_)

PM_10 _particles were collected in an urban environment (EHC-93) and obtained from the Environmental Health Directorate, Health Canada, Ottawa, Ontario. A detailed analysis of the EHC-93 has been presented elsewhere [17]. Particles were suspended at a concentration of 1 mg/ml in hydrocortisone-free supplemented bronchial epithelial cell growth medium (BEGM; Clonetics, San Diego, CA) and sonicated 3 times for 1 min each at maximal power on a Vibra Cell VC-50 sonicator (Sonics and Materials Inc., Danbury, CT) prior to adding to the cells. The endotoxin content of the PM_10 _suspension of 100 μg/ml was 6.4 ± 1.8 EU/ml or less than 3.0 ng/ml [10,13]. This dose of LPS has been shown not to activate either AM or lung epithelial cells to produce cytokines [10].

### Isolation of HBEC and human AM

Bronchial tissue and broncho-alveolar lavage (BAL) fluid was obtained from a total of ten patients who underwent lobectomy or pneumonectomy for small peripheral nodules at St. Paul's Hospital, Vancouver. Informed consent was obtained from all subjects and these studies were approved by the Human Ethics Committee of the University of British Columbia. All subjects were current smokers and were asked to abstain from smoking for 6 weeks prior to the operation. Their mean age was 67.2 yr (range 56–74 yr) (6 women and 4 men). Primary HBEC were isolated from bronchial tissues according to a previously described procedure [10]. In brief, pieces of excised human bronchial tissue approximately 1 cm long were incubated at 4°C for 24 h with 0.1% protease (Type14; Sigma) solution prepared in BEGM containing Fungizone (1 μg/ml; GIBCO BRL, Gaithersburg, MD). The epithelial cells were harvested, washed with BEGM with added antibiotics(100 U/ml of penicillin and 100 μ/gml of streptomycin; Sigma)and Fungizone, and cultured in a 25-cm^2 ^cell culture flask until 80 to 90% confluent. Then the cells were trypsinized and placed in 100-mm cell culture dishes and cultured in BEGM. Light microscopy showed that 95% of the isolated cells had features of bronchial epithelial cells, that is they formed a monolayer of ciliated cells. Also, by trypan blue exclusion, >95% of these cells were viable. Human AM were harvested from BAL fluid obtained from lung segments or lobes that were free of the tumor using a method previously described in detail [7,13]. The BAL fluid cells were >90% viable (trypan blue exclusion method) and consisted of 90–95% AM (as assessed by Wrights-Giemsa stain) and less than 2% neutrophils. AM mono-cultures and AM/HBEC co-cultures were suspended in BEGM. BEGM used throughout this study was without hydrocortisone.

### Exposure of cells to PM_10_

Primary HBEC from the third or fourth passage of cells from each patient were cultured to 90–100 % confluence in 100-mm cell culture dishes (approximately 2.5–3.0 × 10^6 ^cells/dish) then exposed for 2 and 24 h to fresh stock suspensions of 100 μg/ml PM_10 _(EHC-93) prepared in BEGM.

AM (1.0 × 10^7^) from each patient were placed in 100-mm cell culture dishes and allowed to adhere to the plastic dish for 30 min in humidified incubator (5% CO_2 _at 37°C). The non-adherent cells less than 1.0 × 10^6^) were then removed by rinsing twice with BEGM and adherent AM (>98% AM) were incubated in 10 ml of BEGM with or without 100 μg/ml of PM_10 _for 2 and 24 h.

In co-culture experiments, freshly prepared AM (5.0 × 10^6^) were directly placed on the confluent HBEC monolayers which were grown in 100-mm cell culture dishes. The AM were allowed to adhere to HBEC and the non-adherent cells were removed by washing twice with BEGM. The AM/HBEC co-cultured cells were incubated in 10 ml of BEGM with or without 100 μg/ml of PM_10 _for 2 and 24 h. Cell viability was determined following the 24 h PM_10 _exposure in all experiments using the trypan blue exclusion method.

### RNase protection assay (RPA)

After 2 or 24 h treatment, total RNA was isolated from the cells using a single-step phenol/chloroform extraction procedure (Trizol, Life Technologies, Inc., Grand Island, NY). The levels of inflammatory mediator mRNA were determined using the RiboQuant™ multi-probe system (PharMingen, San Diego, CA) following the instructions of the supplier. Two customized template sets were used that included mRNAs of the following inflammatory mediators: human regulated on activation, normal T-cells expressed and secreted (RANTES), macrophage inflammatory protein (MIP)-1β, granulocyte-macrophage colony-stimulating factor (GM-CSF), M-CSF, monocyte chemotactic protein (MCP)-1, interleukin (IL)-6 and leukemia inhibitory factor (LIF). Human ICAM-1 mRNA was determined using a separate template set. Internal controls included mRNAs of the ribosomal protein L32 and glyceraldehyde-3-phosphate dehydrogenase (GAPDH). In brief, 10 μg of total cellular RNA was hybridized overnight to the [α-^32^P] UTP-labeled riboprobes which had been synthesized from the supplied template sets. Single-stranded RNA and free probe remaining after hybridization were digested by a mixture of RNase A and T1. The protected RNA was then phenolized, precipitated, and analyzed on a 5% denaturing polyacrylamide gel. Following electrophoresis, the gel was dried under vacuum and subjected to autoradiography. The quantity of protected labeled RNA was determined using densitometry and the NIH image 1.63 software (National Institutes of Health, Bethesda, MD). Results were normalized to the expression of the internal control, GAPDH. For the densitometric analysis each RPA was repeated four to six times.

### ELISA measurements

Cell culture supernatants were collected 24 h after addition of 100 μg/ml of PM_10 _suspension, centrifuged, filtered through a syringe filter with pore size of 0.22 μm (Corning, Cambridge, MA) to eliminate as much as possible any remaining particles and stored at -80°C until use. MIP-1β, GM-CSF, M-CSF, MCP-1 and IL-6 levels were measured by the Cytokine Core Laboratory (Baltimore, MD) using an ELISA based on a biotin-strepavidin-peroxidase detection system as previously described [10]. All measurements were done in triplicate and values corrected for the number of AM used in each experiment are reported as the means of five experiments.

### Immunocytochemistry

To demonstrate cell surface ICAM-1 (CD54) expression on HBEC and CD11b on AM, cells were placed or grown on coverslips in 6-well plates and incubated for 2 or 24 h with 100 μg/ml of PM_10_. Cells were fixed with 1% paraformaldehyde for 10 min and immunocytochemistry was performed by the alkaline phosphatase anti-alkaline phosphatase method using mouse anti-human CD54 monoclonal antibody (Immunotech, Marseille, France) and mouse anti-human CD11b monoclonal antibody (DAKO, Copenhagen, Denmark) to identify cell surface expression of ICAM-1 and CD11b.

### Cell adhesion blockers and ICAM-1 cross-linking

In experiments testing whether anti-CD54 and anti-CD11b block mediator production by co-cultured AM/HBEC, HBEC and AM were preincubated for 1 h before PM_10 _exposure with control IgG F(ab')_2 _fragments (2 μg/ml; Jackson ImmunoResearch Laboratories, PA), mouse anti-human monoclonal CD54 F(ab')_2 _fragments, and/or monoclonal CD11b F(ab')_2 _fragments (1 μg/ml, respectively; Caltag Laboratories, CA). Cells were then co-cultured and exposed to PM_10 _for 24 h in the presence of the blocking antibodies before analysis by RPA. To determine whether ligand binding to CD54 on HBEC in of itself contributes to the enhanced mediator response of these cells to PM_10 _stimulation cross-linking antibodies to CD54 were used to simulate this possibility. We used previously reported methods of cross-linking CD54 which resulted in intracellular signaling [18,19]. After 2 h of exposure to PM_10_, HBEC were incubated for 1 h with 1 μg/ml mouse anti-human CD54 or 1 μg/ml control mouse non-specific IgG (DAKO). Cells were washed and then incubated for 4 h with 10 μg/ml rabbit anti-mouse IgG (DAKO) to cross-link the bound anti-CD54 and mRNA mediator expression was assessed as above.

### Statistical Analysis

Data are expressed as mean values ± SE. The minimum number of replicates for all measurement was at least three. For RPA and ELISA, differences between matched pairs (control versus PM_10 _treated) were compared by Wilcoxon signed ranked test. To compare mediator production by co-cultures to that by AM plus HBEC mono-cultures, we used the Mann-Whitney *U *test. Differences between multiple groups were compared by one-way analysis of variance (ANOVA). The post hoc test for multiple comparisons was the Dunnett's test. Significance was assumed at p < 0.05.

## Results

### AM/HBEC co-cultures and PM_10_

We previously showed that the majority of AM and HBEC were in contact with each other in our co-culture system [13]. Both cells internalized PM_10 _particles with many cells containing more than one particle. The 100 μg/ml concentration of PM_10 _used throughout this experiment was not toxic to either AM or HBEC and >90% of cells were viable after 24 h exposure as assessed by the trypan blue exclusion method.

### Expression of mRNA induced by PM_10_

**Figure 1:**
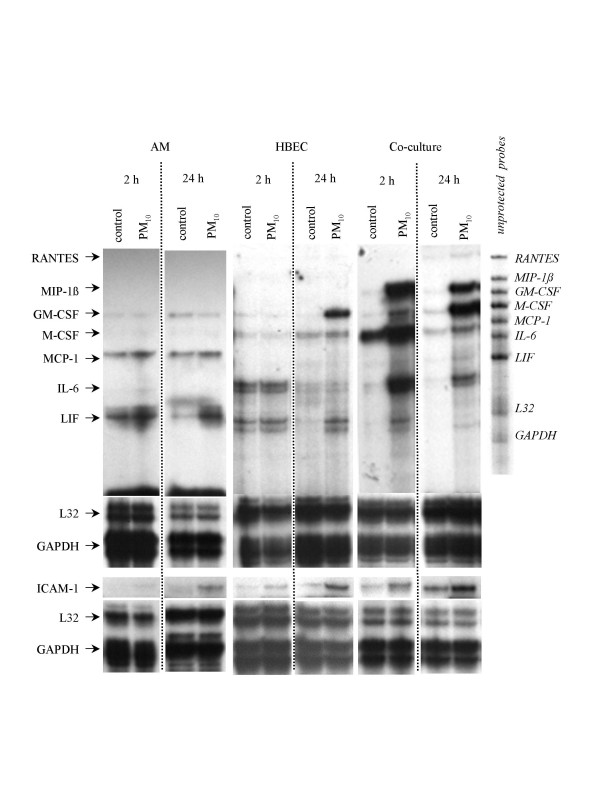
**RNase protection assay of mRNA expression by AM and HBEC**. Representative autoradiographs of RNase protection assays (RPAs) showing mediator expression by AM/HBEC co-cultures, AM mono-cultures and HBEC mono-cultures after 2 and 24 h incubation in medium alone (control) or a 100 μg/ml of PM_10 _suspension (PM_10_). After 24 h exposure AM showed increased expression of LIF and ICAM-1 mRNA. Expression of GM-CSF, LIF and ICAM-1 mRNA by HBEC was increased by 24 h PM_10 _stimulation compared to their respective controls. MIP-1β, GM-CSF, M-CSF, MCP-1, IL-6, LIF and ICAM-1 mRNA expression by AM/HBEC co-cultures was increased 2 and/or 24 h after incubation with PM_10 _compared to control. L32 and GAPDH were used as controls for lane loading.

**Figure 2:**
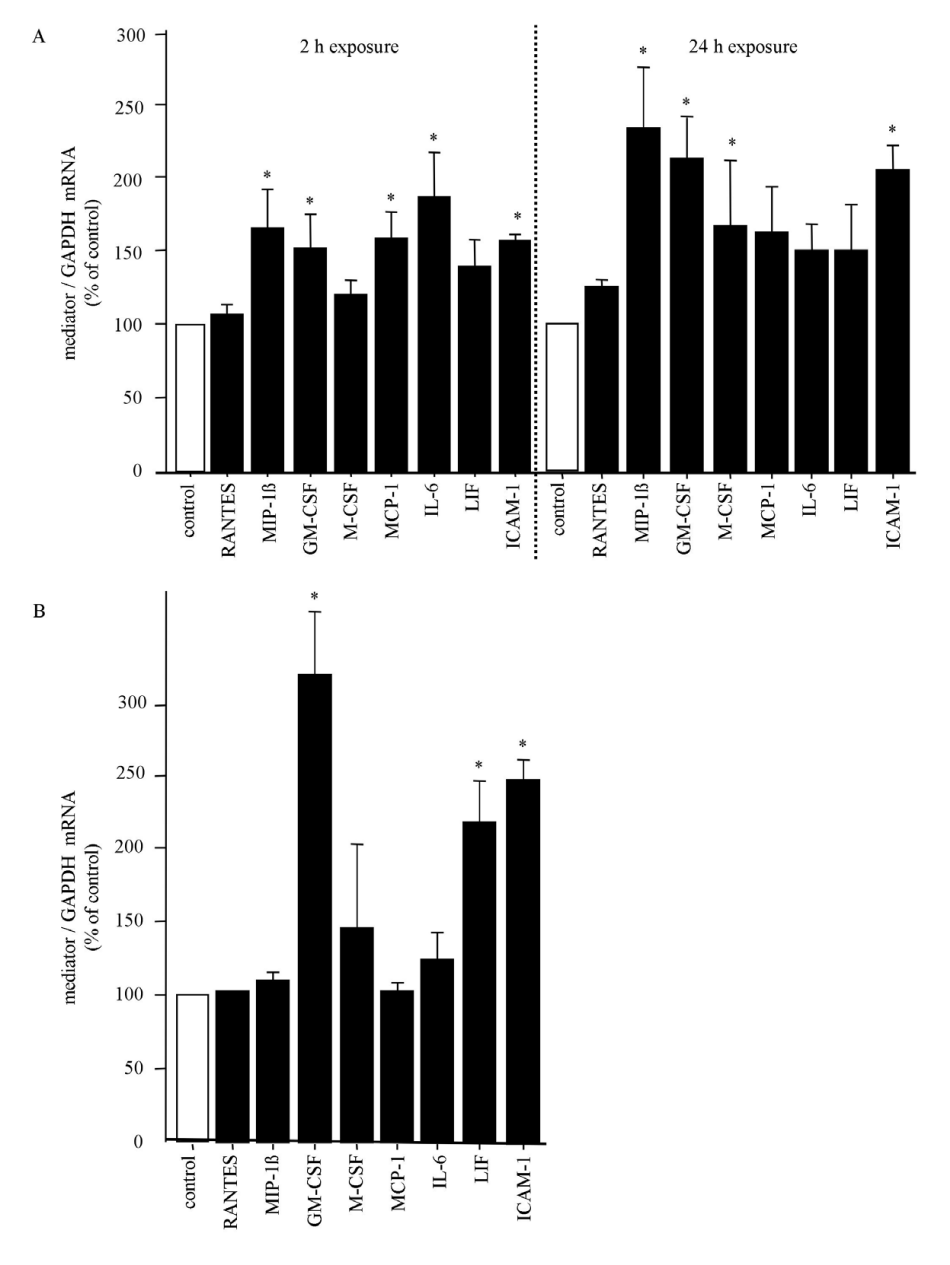
**Densitometric analysis of bands on RPAs**. (A): the density of the bands representing the mediator mRNAs in AM/HBEC co-cultures on autoradiographs such as that shown in Figure 1A was compared to that of the GAPDH mRNA band in the same lane and the resulting ratio (PM_10_; *black bars*) is shown as the percentage change from control values (*white bars*). The mean densitometric value confirmed that the mRNA levels of MIP-1β, GM-CSF, MCP-1, IL-6 and ICAM-1 at 2 h and those of MIP-1β, GM-CSF, M-CSF and ICAM-1 after 24 h exposure were significantly higher than control values. Values are means ± SE of six experiments representing the AM/HBEC co-culture group. (B): the mean densitometric value confirmed that the mRNA levels of GM-CSF, LIF and ICAM-1 at 24 h exposure were significantly higher than control values. Values are means ± SE of four experiments representing the HBEC mono-culture group. *p < 0.05 compared with control.

Representative autoradiographs of mRNA expression by AM or HBEC mono-cultures and AM/HBEC co-cultures after 2 and 24 h incubation in medium alone (control) or a 100 μg/ml of PM_10 _suspension (PM_10_) are shown in Figure 1. Because the RPA kit is not provided with an internal control to account for variation between autoradiographs of different pairs of control versus PM_10 _treated cells such as those shown in Figure 1 and due to that fact that cells from a single but different patient are represented in each different pair, as documented by the differences in intensity of the control L32 band(s) compared to that of the corresponding GAPDH bands as well as differences in the L32 banding pattern (Fig. 1), expected large variations in densitometric data between corresponding pairs of autoradiographs were found. Despite these variations the compiled densitometric analyses of these autoradiographs yielded statistically significant results. However, because of the unavoidable variations, the compiled densitometric results for a few mediators differed from that depicted in the representative autoradiographs. In Figure 1 mRNA expression of the inflammatory mediators of interest, RANTES, MIP-1β, GM-CSF, M-CSF, MCP-1, IL-6 and LIF, was not altered after 2 h of PM_10 _exposure of neither AM nor HBEC mono-cultures and this result was confirmed after densitometric analysis (n = 4, data not shown). Only the expression of ICAM-1 mRNA by HBEC at this time-point appears to be marginally increased (Fig. 1) but after densitometric analysis this change was not found to be significant (n = 4, data not shown). In contrast to the results from the mono-cultures, PM_10 _exposure for 2 h of co-cultured AM/HBEC increased MIP-1β, GM-CSF, M-CSF, IL-6, LIF and ICAM-1 mRNA expression (Fig. 1) and, of these, densitometric analysis of six such RPA experiments confirmed that increases in MIP-1β, GM-CSF, IL-6, and ICAM-1 were significant (Fig. 2A), as well as that of MCP-1 (Fig. 2A) which was not detected in the representative autoradiograph (Fig. 1).

After 24 h exposure to PM_10 _the representative autoradiographs showed that increases in mRNA expression by AM mono-cultures were restricted to that of LIF and ICAM-1 (Fig. 1) but this was not confirmed after statistical analysis of the densitometric results (n = 4, data not shown). In contrast, the increases in GM-CSF, LIF and ICAM-1 by HBEC mono-cultures (Fig. 1) were found to be statistically significant (p < 0.05 and n = 4, respectively)(Fig. 2B). Co-cultures exposed to PM_10 _at this time-point showed strong increases in MIP-1β, GM-CSF, M-CSF, IL-6 and ICAM-1 mRNA and minor increases in those of MCP-1 and LIF (Fig. 1). Except for IL-6, the strong increases were confirmed by the densitometric analysis of six RPA experiments (Fig. 2A).

### Mediator production induced by PM_10_

**Figure 3:**
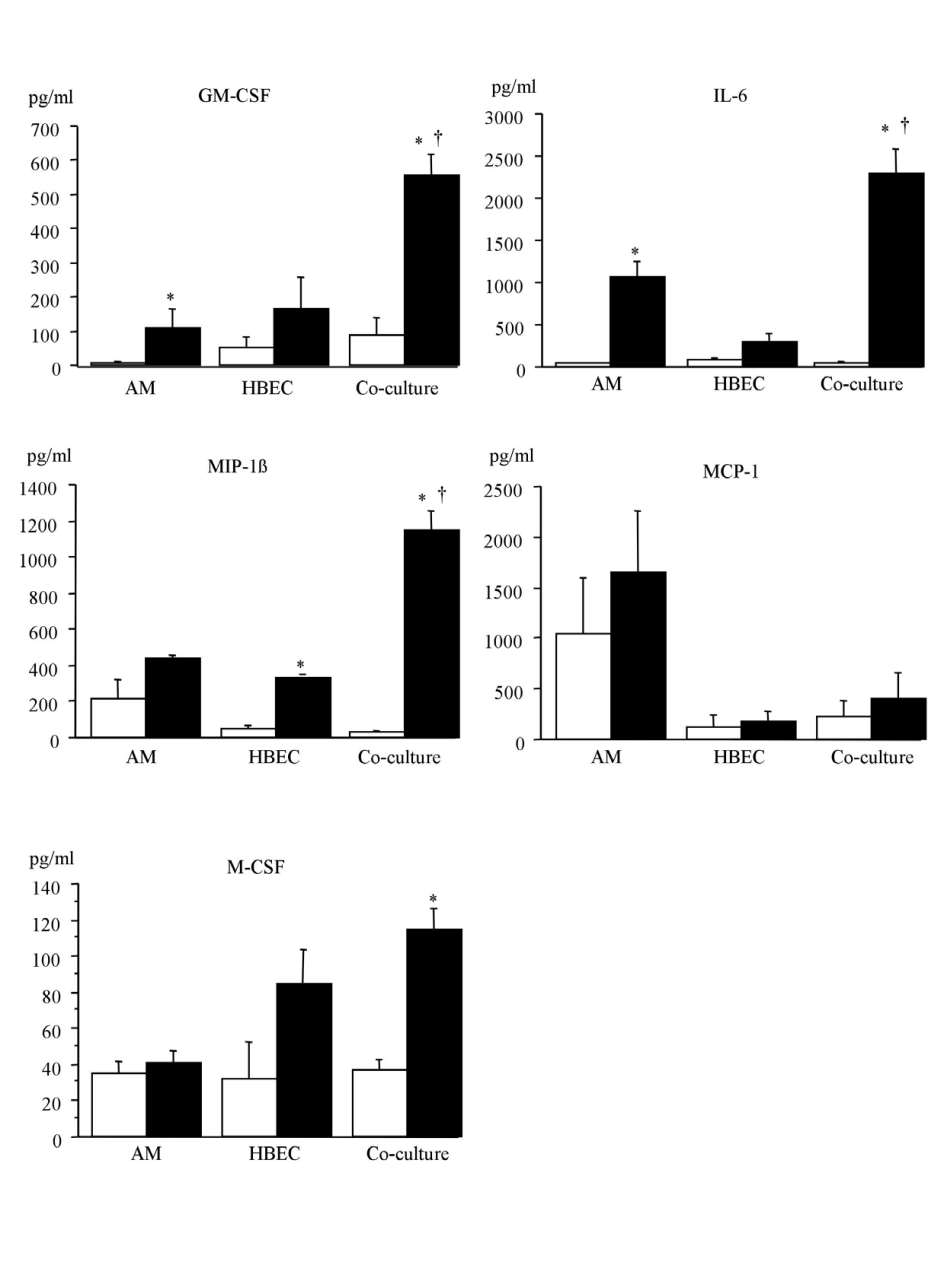
**Mediator protein levels in supernatants of AM and HBEC**. GM-CSF, IL-6, MIP-1β, MCP-1, and M-CSF protein levels in supernatants of AM mono-cultures, HBEC mono-cultures and AM/HBEC co-cultures incubated for 24 h with medium alone (control; *white bars*) or 100 μg/ml of PM_10 _(*black bars*). GM-CSF and IL-6 production by AM mono-cultures and AM/HBEC co-cultures stimulated by PM_10 _increased significantly compared to controls. Exposure to PM_10 _also increased MIP-1β production by HBEC mono-cultures and AM/HBEC co-cultures and M-CSF production by AM/HBEC co-cultures. The GM-CSF, IL-6 and MIP-1β produced by exposed AM/HBEC co-cultures significantly exceeded the sum of those produced by AM and HBEC mono-cultures exposed separately. Values are means ± SE of five experiments. * *p *< 0.05 compared with control. † *p *< 0.05 for exposed AM/HBEC co-cultures compared to the sum of the exposed HBEC and AM mono-cultures.

Figure 3 shows the GM-CSF, IL-6, MIP-1β, MCP-1 and M-CSF protein levels in supernatants of AM/HBEC co-cultures, AM mono-cultures and HBEC mono-cultures incubated for 24 h with medium alone (control) or with 100 μg/ml of PM_10_. GM-CSF, IL-6, MIP-1β and M-CSF production by AM/HBEC co-cultures stimulated by PM_10 _were significantly increased compared to control levels. GM-CSF and IL-6 production by AM mono-cultures stimulated with PM_10 _suspension increased significantly compared to controls. MIP-1β production by HBEC mono-culture stimulated by PM_10 _were significantly increased over control levels.

The GM-CSF, IL-6 and MIP-1β produced by AM/HBEC co-cultures in response to PM_10 _stimulation were more than the sum of the respective mediator produced by PM_10 _exposed AM and HBEC mono-cultures alone suggesting a synergistic effect in production of these cytokines (p < 0.05). This synergistic effect was not seen in the production of M-CSF. MCP-1 production was not significantly increased by PM_10 _in either mono-cultures or AM/HBEC co-cultures but its expression by AM tended to be decreased by co-culturing (Fig. 3).

### Expression of ICAM-1 induced by PM_10_

**Figure 4:**
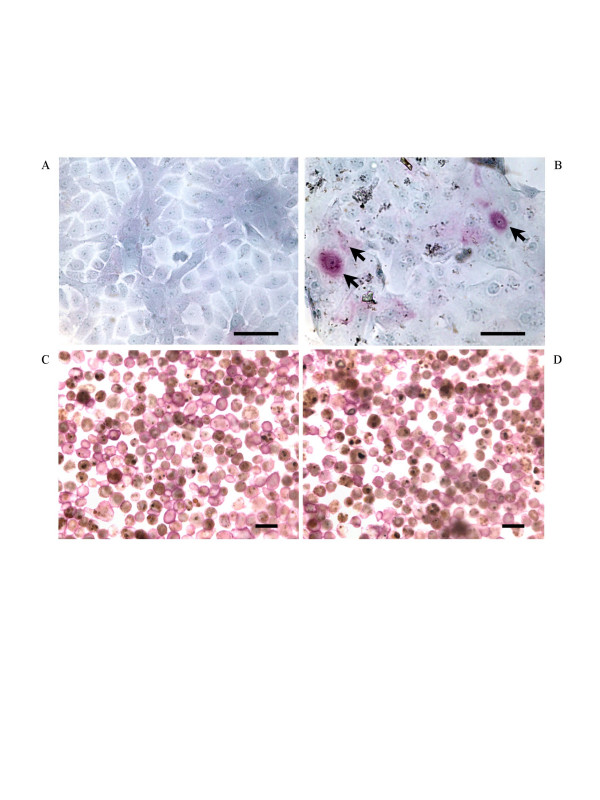
**Surface expression of ICAM-1 on HBEC and CD11b on AM**. Photomicrographs of primary cultured HBEC and human AM on coverslips. Immunocytochemistry was performed using mouse anti-human CD54 monoclonal antibody on HBEC and mouse anti-human CD11b monoclonal antibody on AM. In the absence of PM_10 _stimulation HBEC rarely expressed CD54 (A). After stimulation with 100 μg/ml of PM_10 _for 24 h the majority of cells stained positively (arrows, pink cells) for CD54 (B). Expression of surface CD11b on AM (C) was unaffected by 2 h stimulation with PM_10 _(D). The scale bars represent 20 μm.

Figure 4 shows immunocytochemically stained CD54 on HBEC (to identify ICAM-1) and CD11b on AM. In the absence of PM_10_, HBEC express low levels of ICAM-1 on their cell surface (Fig. 4A). After stimulation with 100 μg/ml of PM_10 _for 24 h many more cells stained positively for ICAM-1 and their intensity of staining was increased (Fig. 4B). Most AM expressed surface CD11b and this expression was unaffected by 2 and 24 h stimulation with PM_10 _(Fig. 4C, D and data not shown).

### ICAM-1 and PM_10_-induced mediator production by AM/HBEC co-cultures

**Figure 5:**
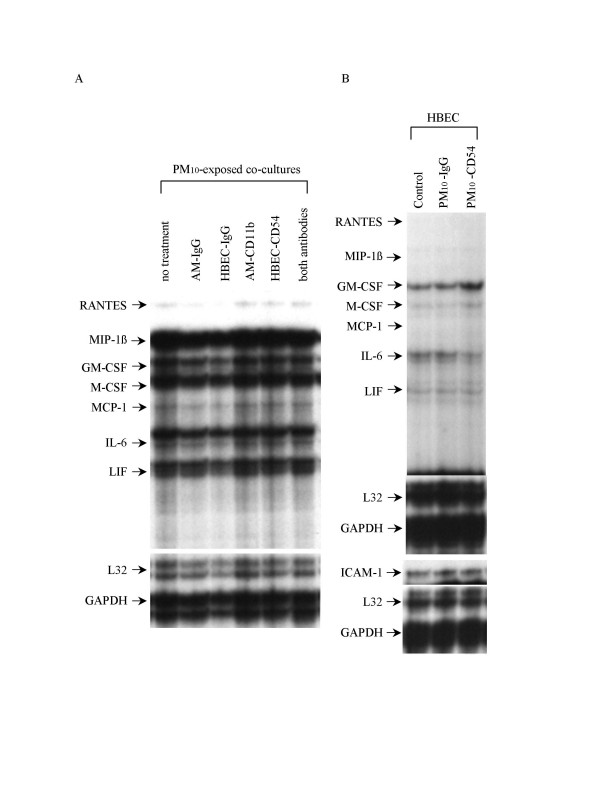
**AM/HBEC co-culture responses after pretreatment with cell adhesion blockers**. (A): autoradiographs from RNase protection assay of mediator mRNA expression by AM/HBEC co-cultures pretreated before 24 h incubation in a 100 μg/ml of PM_10 _suspension including no pretreatment (no treatment) before co-culture, AM pretreated with control IgG (AM-IgG), HBEC with control IgG (HBEC-IgG), AM with anti-CD11b antibody (AM-CD11b), HBEC with anti-CD54 antibody (HBEC-CD54) and both cell types with respective antibodies (both antibodies). The mRNA expression in PM_10_-exposed AM/HBEC co-cultures was not affected by any pretreatments with these antibodies. (B): in the absence of AM, pretreatment of HBEC to cross-link CD54 with antibody followed by 2 h exposure to PM_10 _(PM_10_-CD54) did not alter mediator expression compared with HBEC pretreated with control IgG (PM_10_-IgG) and non-pretreated HBEC (control).

To determine the role of β2-integrin/ICAM-1 interaction in mediator production by AM/HBEC co-cultures, AM and HBEC were incubated with inhibitors of these adhesion molecules before PM_10 _exposure. Representative autoradiographs of mRNA expression by such AM/HBEC co-cultures after 24 h incubation in a 100 μg/ml of PM_10 _suspension are shown in Figure 5A. They include pretreatment of neither cell before co-culture, of only AM with control IgG or anti-CD11b antibody, of only HBEC with control IgG or anti-CD54 antibody, and of both cell types with both antibodies. The increased mRNA expression in PM_10_-stimulated AM/HBEC co-cultures was not affected by any of the pretreatments with these antibodies. In addition, as shown in Figure 5B, CD54 cross-linking itself in the absence of AM did not induce mediator expression in HBEC exposed to PM_10_.

## Discussion

AM and lung epithelial cells play a key role in processing inhaled particulate matter. In the present study we confirmed that exposing co-cultures of human AM and HBEC atmospheric particles to for 2 hr increased mRNA expression of GM-CSF, MCP-1 and IL-6 [13]. The current addition of mRNAs, that of M-CSF and MIP-1β, to this list of these mediators involved in the marrow production, mobilization and recruitment of monocytes that are increased in response to PM_10 _exposure reinforces the hypothesis that exposure of the lung to environmental pollutants can stimulate a systemic inflammatory response [4]. Besides these bone marrow oriented mediators, mRNA expression of ICAM-1, an adhesion molecule potentially involved in an interaction between AM and HBEC to amplify marrow-related mediator expression, was increased. Another important hitherto unreported finding, that of sustained increased expression of many of these mediator mRNAs, MIP-1β, GM-CSF, M-CSF, and ICAM-1, over 24 h of exposure, supports the robust increase in the expression of the corresponding mediator proteins that we observed. These included MIP-1β, M-CSF, ICAM-1 as well as the previously reported GM-CSF and IL-6 [13]. Furthermore, the synergistic increases in GM-CSF, IL-6 and MIP-1β secretion by the co-cultures compared to the sum of the mono-cultures in response to PM_10 _exposure indicate an interaction between these cells with ICAM-1 possibly contributing to this interaction.

IL-6, the hematopoietic growth factors GM-CSF and M-CSF, and the C-C chemokine MIP-1 are important mediators in the production and mobilization of monocytes from the bone marrow [20-22]. IL-6 is considered an important multifunctional cytokine involved in the regulation of a variety of cellular responses, including being a permissive factor for monocytic colony formation by human hematopoietic progenitor cells in combination with GM-CSF [23]. Monocytes recruited into the lung play a critical important role in clearing foreign material such as particles from the lung which underscores the importance of mediators such as GM-CSF as both a pro-inflammatory but also an anti-inflammatory mediator. This anti-inflammatory role is supported by studies that showed that GM-CSF has a protective role against pulmonary fibrosis [24] or hyperoxic lung injury [25] in animal models. Both IL-6 and GM-CSF stimulate the marrow to produce and release monocytes while the acute response cytokines, IL-1 and TNF-α, secreted in response to PM_10 _stimulation by AM [7,13] induce the production of monocytic chemoattractants such as MCP-1 [20,21,26-29]. MIP-1β is a chemotactic factor for human monocytes similar to MIP-1α [22]. Because PM_10 _did not induce MIP-1β production in human AM [4] or its mRNA in HBEC in the current study, increased MIP-1β expression in the co-cultures most likely relies on an interaction between these two cells. The significance of such an interaction is reinforced by our finding that the production of this chemokine in response to PM_10_, along with that of GM-CSF and IL-6, is synergistically increased, as noted above, when AM and HBEC are co-cultured. Such a synergistic increase in mediator production could augment the release of both monocytes and polymorphonuclear leukocytes from the bone marrow observed after stimulation by mediators produced by AM incubated alone with EHC-93 *ex vivo *[6] and thus contribute to a similar response to *in vivo *exposure to the ambient particles [6,15].

MCP-1 was the other C-C chemokine that we studied. Along with additional support from results from our laboratory [15], Rosseau and colleagues [30] have shown that the induction of MCP-1 in AM is a major contributor to the recruitment of peripheral blood monocytes into the alveolar compartment. In the present study we showed that production of MCP-1 by AM was just marginally increased by PM_10 _exposure (p = 0.07). Interestingly, the production and release of MCP-1 by AM/HBEC co-cultures tended (not significant) to be lower than by AM alone (Fig. 3). In AM/HBEC co-cultures, expression of MCP-1 mRNA was significantly increased by PM_10 _after 2 h but not 24 h exposure suggesting suppression of MCP-1 expression following prolonged exposure of lung cells to particles. This suggests a translational or post-translational control of MCP-1 production and could be an important immunomodulatory pathway by which the local inflammatory reaction in the lung is controlled after PM_10 _exposure. Together, our findings suggest that both colony stimulating factors and chemokines are released from lung cells following the inhalation of atmospheric particles and that these mediators are critically important in the production and the release of monocytes from the marrow as well as their recruitment into the lung.

The close proximity of AM and epithelial cells in the lung suggests that interaction between these cells is critically important in generating inflammatory mediators in response to noxious stimuli. Previous studies from our laboratory [13] support this concept showing that AM and epithelial cells in co-culture interact to amplify their pro-inflammatory mediator mRNA generation in response to PM_10 _exposure compared to exposure of mono-cultures of these cells. That soluble factors contribute to this interaction was shown when conditioned media from PM_10_-stimulated AM induced increases in mRNA expression of many of these mediators in HBEC [13]. On the other hand, that cellular contact between different lung cells (e.g., epithelial, endothelial cells, and fibroblast) is necessary for cell activation and cytokine production [11,31] has also been demonstrated. Along these lines, we recently showed increased expression of ICAM-1 mRNA after incubation of HBEC with conditioned media from PM_10_-stimulated AM and this response was blocked by neutralizing antibodies to TNF-α and IL-1β [7]. While TNF-α and IL-1β appear to be major players in the interaction between AM and HBEC in response to PM_10_, our results suggest that ICAM-1 may play an important role in facilitating the AM-HBEC interaction via these soluble factors.

We postulated that adhesive interactions between CD11/CD18 on AM with ICAM-1 on HBEC contribute to the amplified production of cytokines from AM/HBEC co-cultures observed in the current study. Previous studies have demonstrated that cross-linking CD11b/CD18 on the surface of phagocytes using a combination of either its ligand ICAM-1 or anti-ICAM-1 antibodies primes phagocytes for increased respiratory burst and release of reactive oxygen intermediates [32-34]. In the present study we showed increased epithelial cell surface expression of ICAM-1 induced by PM_10 _exposure, while CD11b was constitutively expressed on the surface of AM. Cross-linking ICAM-1 on HBEC did not change their PM_10_-induced mRNA expression. Furthermore, blocking CD11b/CD18 and one of its ligands, ICAM-1 (CD54), did not block or decrease the PM_10_-induced mRNA expression in AM/HBEC co-cultures. These results are consistent with those of Tao and co-workers [35] who demonstrated that TNF-α and MIP-2 responses to urban air particles in rat AM and RLE (rat alveolar type II epithelial cell line) co-cultures were not blocked with anti-CD18 (β2-integrins)/CD54, arginine-glycine-aspartate peptide (against β1/β3-integrins) and heparin (non-specific anti-inflammatory agent). Paine and colleagues [36] demonstrated that blocking ICAM-1 (anti-CD54 F(ab')_2 _fragments) decreased rat AM phagocytosis of beads and their planar chemotaxis over the surface of rat alveolar type I epithelial cells. This suggests that ICAM-1 is important for the efficient phagocytosis of particles by AM and promotes mobility of AM on airway epithelial cell surface in the alveolus. Together these studies showed that the β2-integrin/ICAM-1 interaction between AM and lung epithelial cells are important in the chemotaxis of AM in the lung and their phagocytosis of inhaled particles but that this adhesive interaction may not contribute to the mediator production and release by AM and lung epithelial cells. These findings do not exclude the possibility that other adhesive interactions or simultaneous adhesive interactions of more that one adhesion molecule are involved in the particle-induced AM-bronchial epithelial cell mediator response.

## Conclusion

Exposure of AM/HBEC co-cultures to ambient particles increased the expression and release of a variety of inflammatory mediators including GM-CSF, M-CSF, IL-6 and MIP-1β that enhance bone marrow production of monocytes and their recruitment into the lung. In addition this type of exposure resulted in synergistic production of GM-CSF and IL-6 in the co cultured cells. The adhesive interaction between ICAM-1 on epithelial cells with the β2-integrin CD11b on AM did not contribute to this synergistic mediator production. We speculate that the interaction between AM and lung epithelial cells amplifies PM_10_-induced lung inflammation and contributes to the pulmonary morbidity associated with exposure to particulate matter air pollution. This enhanced lung inflammation may also contribute to the systemic inflammatory response as well as the cardiovascular morbidity and mortality induced by air pollution [1,2,37].

## Authors' contributions

HI carried out all through the experiments and drafted the manuscript. TF, YG, NS and HM participated in the design of the study. SH and JCH participated in its design and helped to draft the manuscript. SFVE conceived of the study, participated in its design and coordination and helped to draft the manuscript. RV provided EHC-93. All authors read and approved the final manuscript.
